# Intelligence

**Published:** 1810-03

**Authors:** 


### INTELLIGENCE.

### Medical Society of Paris.

This learned body held its first meeting for the present Session, (1809-10) on the 31st of October last.

After a short preliminary discourse by the president, M. Sedillot, the Secretary, in recapitulating the proceedings of the Society during the last year, gave a copious account of the perforation of the tympanum, as having been practised with success in several cases of deafness.

M. Nacquart read a paper under the title of "Topographical and Medical Observations on the Seventh Municipal Division of Paris." The public utility likely to be derived from a similar work on the forty-seven other municipal sections of Paris, formed the chief theme of this discourse, and M. Nacquart, in laying his own Memoir before the Society, expresses his hope that his example will be followed by the rest of the medical officers of Paris, as well as of other great cities.

The Society afterwards proceeded to award the prizes of the year.

M. François Hebreart, M. D. Surgeon *en second* to the Bicetre, obtained a gold medal of the value of 300 francs for the best Memoir on the following subject, as given out the preceding year: "Describe the character, causes, and treatment of gangrene, considered particularly with regard to the soft parts."

Silver

Silver medals for various valuable communications were respectively voted to M. Thiebault, Physician at Bruxeres; M. Boucher, Surgeon at La Fleche; M. Chapp, Officer of the Legion of Honour, and Surgeon Major to the Imperial Guards; M. Labonnardiere, Physician at Cremieu; and to M. Bertrand of Pont-du Chateau.

The following subject was then announced as a prize question to be decided in October, 1810.

"Describe the disease designated by the ancients, and particularly by the English medical writers, under the name of Angina Pectoris.

"Point out the causes which produce it, and the authors who speak of it in a particular manner; describe the diseases which resemble it most, the affections with which it is complicated, and those which it produces in its turn."

The prize is a gold medal, of the value of 300 Francs. Another gold medal, of the value of 500 Francs, to be awarded in October 1811, has also been offered by the Society for the best paper on the following subject.

"A clear and precise idea is to be given of the nature of contagion. Point out the differences considered with regard to the nature of its principles, and the various modes of its communication. Designate from among the diseases generally considered as contagious, such as are really of that description. Lastly, Point out the mode in which the contagion is communicated in each of them."

An anonymous friend to humanity has presented 300 francs to the Society to be also adjudged at its first meeting in October 1811, to the author of the most satisfactory essay on the following subjects.

"1. Enquire, from direct observations, into the nature and effects of Plica Polonica; point out the epiphenomena as well as any concomitant diseases which are foreign to it; describe the true causes of this affection of the hairy parts of men and animals since its origin, and in the different countries where it has been observed.

"2. Determine the circumstances under which these causes exert their influence; ascertain why the plica has at all times spared or afflicted certain classes, and why, on some occasions, it has been observed to prevail and to disappear alternately.

"3. Appreciate the value of the therapeutic and hygiene means hitherto proposed to combat or prevent this species of endemic; and point out the best method of extirpating it."

The memoirs on any of the above subjects, writen in Latin or in French, may be addressed to M. Sedillot, the Secretary, at No. 6, Rue de Favart. They must reach Paris at least two months previous to the October meeting of the Society, and are to be accompanied with a sealed letter, containing the name of the author in the usual manner.

M. Tarenne has discovered, that the slimy juice of snails is a specific for the cure of hernia, when the ruptured part can be returned, and it is not dangerous to confine it in the body. When this point is ascertained, he directs that a truss be made, having the ball at the end concave instead of convex, as usual, for the reception of a cup of equal diameter with the orifice of the hernia. The cup must be of porcelain, glass, or earthenware, that the liquor may not penetrate it, or undergo any alteration; and the edges of it should be turned, that they may not incommode the patient. It is to be filled with wool, which must be changed every other day. Two, three, or four hundred snails, are then to be procured, and kept in a place where they can procure food, as only two or three, or if they are small, six or eight, are to be used every day. The patient, before he rises, and after he has been in bed, removes the cup from the truss, and pricks the snail in different places with a pin. From each wound the snail gives out, through the opening in his shell, sometimes a bluish, sometimes a grey liquid, which must be caught on the wool in the cup. If only a thick froth oozes out, the snail must be thrown aside, and another taken in his stead. The cup being sufficiently filled with liquor, must always be placed exactly in the same situation on the affected part, then covered with a white linen cloth, and the ball of the truss applied on it. The latter must be sufficiently tight, to prevent the fluid from escaping. During this treatment, which will last three or four months, or more, the only precautions necessary to be taken are to shave the part once in four days, and not to leave it long uncovered, for fear of cold. If the cup rubs off the skin, it must be removed till the place is healed. In this case, the patient may remove the truss altogether at night, if it can be done without danger; and in the day-time he may wear it dry, filling the cavity with wool, and covering the hernia with a bit of cloth. By this treatment, a common hernia may be cured in two, three, or at most four months; after which, however, the patient should continue to wear the truss for six weeks, or two months, till the wound is sufficiently healed, to permit the muscles to resume their natural action.

---

M. Vauquelin, in the name of the Committee of the Chemical Arts, has lately reported on a manufacture of tallow for candles, professed to be purified from all animal substances of an injurious nature, to be free from all moisture, and not at all discoloured. "The tallow," says he, "which I carefully examined, is demi-transparent, perfectly dry, and sonorous. It is indeed so very dry, that when a blade of iron is passed over it, only lightly touching it, it gives an extremely lively phosphoric light, occasioned, according to all appearance, by an electric motion; for when this tallow is recently melted, and the surrounding air is extremely dry, the mere passing of the hand on it is sufficient to produce sparks. The dryness of this tallow is still farther demonstrated monstrated by its perfect transparency when melted: at the temperature of boiling water, neither bubbles nor clouds are discernible. This tallow, it is affirmed, may be kept without any discolouration or rancidity for two years. The candles made of it are extremely white, their light is very pure, they emit little or no smoke; they do not gutter or run, and require snuffing less frequently than others. They are about five per cent. higher in price than those of common manufacture."

---

It has long been matter of surprise to foreign naturalists, that although in this country, botany has been cultivated with a zeal and success which leave nothing to desire, scarcely any attention has been hitherto paid to the sister science entomology; so that while the vegetable productions of the British Isles are for the most part well known, and accurately described, not a third of our numerous tribes of insects have been noticed or enumerated. This neglect is, doubtless, principally to be attributed to the want of a popular and comprehensive elementary work, adapted to the present improved state of the science. To supply this desideratum, and facilitate the study in Britain, of a department of natural history, singularly amusing and instructive, abounding in objects striking in their shape and structure, splendid in decoration, and in the highest degree interesting in habits, manners, and economy, the Rev. W. Kirby, A. B. F. L. S. author of Monographia Apum Angliæ, and Mr. W. Spence, F. L. S, are engaged in preparing an Introduction to Entomology, which is in a state of considerable forwardness. The plan of the work is popular; but without overlooking science, to the technical and anatomical departments of which, much new matter will be contributed. Its object, after obviating objections, and removing prejudices, is to include every thing useful or interesting to the entomological student, except descriptions of genera and species, which are foreign to the nature of such a work.

---

The subject of apartments with a graduated temperature, has for some time attracted the attention of the medical public. We are glad to find, by a paper from Dr. Pearson, in the Philosophical Magazine, that a speculation is on float for erecting spacious buildings on such a plan, to receive those invalids as cannot change their residence to a softer climate. On this occasion we must do justice to our own Journal for having first suggested such a plan. In vol. v. p. 311, is a letter from Dr. Adams to Dr. Willan, giving an account of the climate of Madeira, which he concludes thus. "It may be well worth your while to try whether spacious buildings, regularly heated, safely ventilated, and large enough to admit of necessary exercise, may not answer the purpose for such whose want of means, of courage, or of leisure, prevent their taking a voyage to a more genial climate."

The

The College of Surgeons have at length proceeded so far with their building, under the auspices and by the pecuniary assistance of the nation, that Mr. Hunter's Collection is now under arrangement, at their Museum in Lincoln's Inn Fields, or rather in Portugal Street, for as yet only that side of the building is completed. The theatre is spacious, and well contrived. Sir William Blizard has given his second lecture; and after his course, Mr. Home commences another on Comparative Anatomy.

One of the most remarkable facts in the history of geography, is communicated by letters conveyed in the last ships from the Cape of Good Hope. The island of Bossen, or Penguin, sometimes called Seal Island, at the western extremity of Table Bay, has entirely disappeared beneath the waters. An earthquake was felt at Cape Town, in December, only two leagues distant, by which some damage was occasioned to the houses; but we do not find that any lives were lost at that place; and it is supposed that the convulsion extended to Bossen. The island was about two miles in length and one in breadth, and was, although flat, somewhat more elevated above the surface of the sea, than the contiguous island of Elizabeth. The Dutch, when in possession of the Cape, kept a guard of twenty-four men on Bossen; and it was employed as a place of banishment for criminals, to the number of from seventy to one-hundred, who dug lime-stone to supply materials for the buildings on the adjacent continent. How many lives have been lost by this awful visitation, is not ascertained.

Dr. Adams has nearly completed his preparation for a Course of Lectures on Pathology and Therapeutics. It is his intention to deliver them early in the spring, as soon as the season becomes too much advanced for general dissecting. Further particulars will be given in due time.

Dr. Clough, Physician Man-Midwife to the St. Mary-le-bone General Dispensary, &c. will on Monday the 12th of March, at ten in the morning, commence his next Course of Lectures on the Science of Midwifery, including the Diseases of Women and Infants; and for the convenience of Students attending other Classes, Hospitals, &c. his Evening Course on Tuesday the 13th at seven, at his Lecture Room, 68, Berner's Street. For a Prospectus and Syllabus, apply to Dr. Clough as above.

Dr. Reid will commence his Spring Course of Lectures, on the Theory and Practice of Medicine, on Monday the 19th of March, at six o'clock in the evening, at his house, Grenville Street, Brunswick Square, where the Course will be continued until its conclusion in the latter end of May.

Dr. Latham has in the press, Facts and Opinions concerning Diabetes.

Mr. Lee, surgeon, of Shields, will shortly publish an Essay on Mortification.

Dr. Smith is printing a Translation of Le Roy's Instructions for Gouty and Rheumatic Persons.

A Fourth

A Fourth Edition of Dr. TROTTER'S Essay on Drunkenness, and its Effects on the Human Body, with considerable additions, is now in the press, and will be published in the Course of next month.

Dr. WATSON has nearly ready for publication, a Theoretical and Practical View of the Instruction of the Deaf and Dumb; containing Hints for the Correction of Impediments in Speech, and illustrated by numerous Plates.

We have to lament the early death of a truly valuable member of the profession, Mr. SAUNDERS, of Ely Place, Surgeon to the London Infirmary for Eyes and Ears, at the age of 37. He died somewhat suddenly, but with occasional symptoms which afforded too much probability of mischief in a most important organ. At uncertain intervals, a few weeks before his death, he complained of paralysis in distant parts of his extremities, and sometimes of loss of memory for a short period. On the day of his death he appeared better than usual, till he fell senseless almost before he could express his own sensations. The body was examined by Mr. Astley Cooper and other gentlemen. In the brain, near the pons varolii, was found an abscess, which had gradually extended to an artery, the ulceration of which produced hæmorrhage and immediate death. Due respect was paid to his merits as a scientific character, and the first institutor of a benevolent charity, which through his well directed exertions, aided by his coadjutor Dr. Farr, had acquired an extent of patronage which nothing but the general acknowledgement of its usefulness could have produced. He was buried in the vault of his parish church, St. Andrew's, Holborn, and his funeral was publicly attended.

